# Bis(tetra­propyl­ammonium) di-μ_3_-iodido-di-μ_2_-iodido-diiodidodi­pyridine­tetra­copper(I)

**DOI:** 10.1107/S1600536810010202

**Published:** 2010-03-24

**Authors:** Ehsan Jalilian

**Affiliations:** a Department of Environmental and Material Chemistry, Arrhenius Laboratory, Stockholm University, 106 91 Stockholm, Sweden

## Abstract

The title compound, (C_12_H_28_N)_2_[Cu_3.194_I_6_(C_5_H_5_N)_2_] was prepared from reaction of copper powder, copper(I) oxide, hydro­iodic acid, tetra­propyl­ammonium iodide and pyridine under hydro­thermal conditions. In the centrosymmetric Cu_4_I_6_^2−^ anion, one Cu site is in a trigonal-planar coordination while the second Cu site, which is only partially occupied [site occupancy of 0.5968 (16)], is surroundedby three iodine atoms and one pyridine molecule in a distorted tetrahedral coordination.

## Related literature

For further structural motifs and the luminescence properties of copper(I)iodide with substituted pyridine, see Cariati *et al.* (2005 ▶). For the extinction correction, see: Becker & Coppens (1974 ▶). 
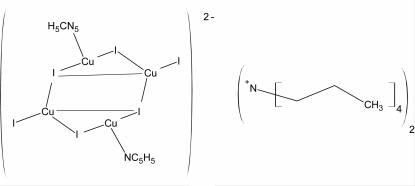


## Experimental

### 

#### Crystal data

(C_12_H_28_N)_2_[Cu_3.194_I_6_(C_5_H_5_N)_2_]
*M* *_r_* = 1495.3
Triclinic, 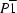

*a* = 8.8974 (2) Å
*b* = 11.8076 (3) Å
*c* = 12.2176 (2) Å
α = 73.2442 (18)°
β = 78.5398 (17)°
γ = 81.4137 (18)°
*V* = 1198.74 (5) Å^3^
*Z* = 1
Mo *K*α radiation
μ = 5.29 mm^−1^
*T* = 100 K
0.47 × 0.17 × 0.13 mm

#### Data collection

Oxford Diffraction Xcalibur3 diffractometer with a Sapphire-3 CCD detector
Absorption correction: Gaussian (*CrysAlis RED*; Oxford Diffraction, 2008 ▶) *T*_min_ = 0.209, *T*_max_ = 0.623
38326 measured reflections
7574 independent reflections
6526 reflections with *I* > 3σ(*I*)
*R*_int_ = 0.020

#### Refinement

*R*[*F* ^2^ > 2σ(*F* ^2^)] = 0.023
*wR*(*F* ^2^) = 0.068
*S* = 1.02
7574 reflections
219 parameters
H-atom parameters constrained
Δρ_max_ = 1.05 e Å^−3^
Δρ_min_ = −0.53 e Å^−3^

### 

Data collection: *CrysAlis CCD* (Oxford Diffraction, 2008 ▶); cell refinement: *CrysAlis RED* (Oxford Diffraction, 2008 ▶); data reduction: *CrysAlis RED*; program(s) used to solve structure: *SUPERFLIP* (Oszlányi & Sütő, 2004 ▶); program(s) used to refine structure: *JANA2000* (Petříček *et al.*, 2000 ▶); molecular graphics: *DIAMOND* (Brandenburg, 1999 ▶); software used to prepare material for publication: *JANA2000*.

## Supplementary Material

Crystal structure: contains datablocks global, I. DOI: 10.1107/S1600536810010202/jh2137sup1.cif

Structure factors: contains datablocks I. DOI: 10.1107/S1600536810010202/jh2137Isup2.hkl

Additional supplementary materials:  crystallographic information; 3D view; checkCIF report

## Figures and Tables

**Fig. 1.**
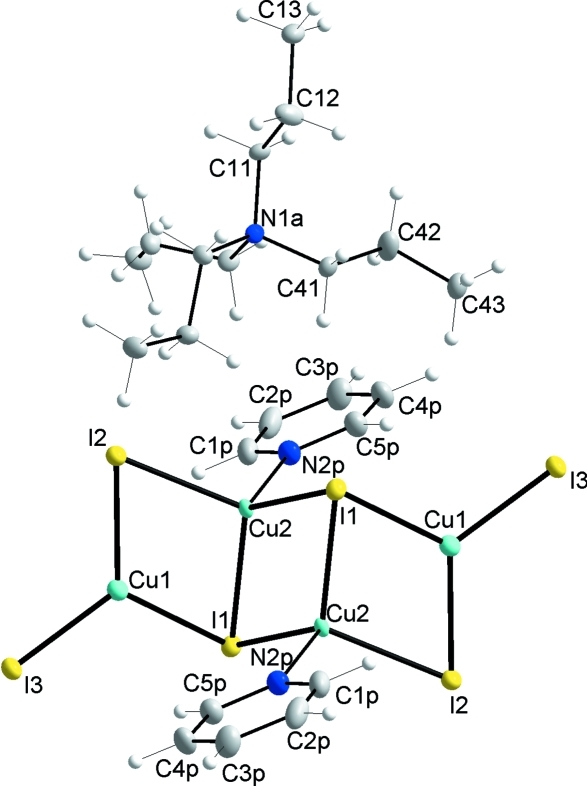
Molecular structure with atom labelling scheme for the I—Cu-Pyridine anion and the tetrapropeylammonium cation in (I). Non H atoms are shown as 50% probability displacement ellipsoids.

## supplementary crystallographic information

### Comment

Due to the wide variation in molecular structure, halocuprates(I) exhibit a very interesting structural chemistry. The most important factor to this structural diversity is the fact that Cu(I) can accept trigonal planar coordination, tetrahedral coordination as well as less defined intermediate arrangements. The further linkage of these units, triangles by corner-sharing or edge-sharing, tetrahedra also by face sharing yields further structural flexibility. Many different version of oligometric and polymeric species in Cu(I)X (X=Cl,Br, I) have been discovered in copper (I) halide based systems containing inflexible N- donor ligands. Stoichiometry and connectivity are dependent on the size and the coordination number of the cation that participate in the structure. The compound presented here 2[(C_12 _H_28 _N)^+^ [I_3_ Cu_1.597_ N C_5_ H_5_]^-^ ] (I), is prepared from reaction between copper powder, copperoxide, tetra n-propylammoniumiodide, pyridine and hydroiodic acid. The anion in (I) has a range of Cu–I distances [ 2.5200 (3)–2.7752 (6) Å] while the I–Cu–I angles spread over large range [108.039 (16)–122.009 (14)°]. One of the two independent Cu positions is clearly under-occupied (Cu2, occ ≈ 0.6). This non-stoichiometry leads to local relaxations that can be seen as anomalously large and anisotropic thermal parameters on the surrounding I neighbours. The compound is light yellow in color, and it is well known that Cu cannot attain the divalent oxidation state in direct contact with iodide, and therefore we conclude that all Cu is in the monovalent state. The charge balance must instead be maintained by the protonation of either the pyridine unit or the Cu_4-x_I_6_ -cluster unit, a likely scenario since the synthesis is run at a low pH value.

The attached pyridine unit is a typical pyridine with N–C and C–C ranges [ 1.345 (3)–1.349 (3) Å and 1.359 (5)–1.398 (4)Å respectively] The angle C–N–C is 119.2 (2)° while (N/)C–C–C the angles ranges [ 118.4 (2)–120.8 (2)°]. The cation is a regular tetra propylammonium ion with N–C and C–C distances range [1.518 (3)–1.521 (3) Å and 1.516 (3)–1.542 (3)Å respectively], and the C–N–C, (N/)C–C–C angels range between [107.99 (16)–111.36 (15)° and 108.40 (19)–115.60 (18)°].

### Experimental

Copper powder (2.854 mmol), copper(I)oxide (2.827 mmol), hydroiodic acid (7.6 mmol) tetra n-propylammonium iodide (3.101 mmol) and pyridine (12.958 mmol) were put into an autoclave and heated at 165 °C for 19 h. It resulted in yellow crystals that luminesce vividly under UV light.

### Refinement

The structures were solved by charge-flipping, giving the I, Cu, P and a major part of the C positions. Subsequently the remaining C positions were found using difference Fourier analysis. All non-hydrogen positions were refined using full matrix least squares. The hydrogen atoms were located by geometrical methods and were allowed to ride, with C–H = 1.00 Å and *U*_eq_ = 1.2*U*_iso_(C).

### Crystal data

**Table:**

(C_12_H_28_N)_2_[Cu_3.194_I_6_(C_5_H_5_N)_2_]	*Z* = 1
*M**_r_* = 1495.3	*F*(000) = 708.8
Triclinic, *P*1	*D*_x_ = 2.071 Mg m^−^^3^
Hall symbol: -P 1	Mo *K*α radiation, λ = 0.71069 Å
*a* = 8.8974 (2) Å	Cell parameters from 27591 reflections
*b* = 11.8076 (3) Å	θ = 4.3–32.2°
*c* = 12.2176 (2) Å	µ = 5.29 mm^−^^1^
α = 73.2442 (18)°	*T* = 100 K
β = 78.5398 (17)°	Rodd, yellow
γ = 81.4137 (18)°	0.47 × 0.17 × 0.13 mm
*V* = 1198.74 (5) Å^3^	

### Data collection

**Table:**

Oxford Diffraction Xcalibur3 diffractometer with a Sapphire-3 CCD detector	7574 independent reflections
Radiation source: Enhance (Mo) X-ray source	6526 reflections with *I* > 3σ(*I*)
graphite	*R*_int_ = 0.020
Detector resolution: 16.5467 pixels mm^-1^	θ_max_ = 32.2°, θ_min_ = 4.3°
ω scans	*h* = −13→13
Absorption correction: gaussian (*CrysAlis RED*; Oxford Diffraction, 2008)	*k* = −17→17
*T*_min_ = 0.209, *T*_max_ = 0.623	*l* = −18→18
38326 measured reflections	

### Refinement

**Table:**

Refinement on *F*^2^	Weighting scheme based on measured s.u.'s *w* = 1/[σ^2^(*I*) + 0.0025*I*^2^]
*R*[*F*^2^ > 2σ(*F*^2^)] = 0.023	(Δ/σ)_max_ = 0.048
*wR*(*F*^2^) = 0.068	Δρ_max_ = 1.05 e Å^−^^3^
*S* = 1.02	Δρ_min_ = −0.53 e Å^−^^3^
7574 reflections	Extinction correction: B-C type 1 Gaussian isotropic (Becker & Coppens, 1974)
219 parameters	Extinction coefficient: 2863
H-atom parameters constrained	

### Fractional atomic coordinates and isotropic or equivalent isotropic displacement parameters (Å^2^)

**Table:**

	*x*	*y*	*z*	*U*_iso_*/*U*_eq_	Occ. (<1)
I1	0.975221 (18)	0.302248 (12)	0.020355 (12)	0.01742 (5)	
I2	0.969321 (19)	0.432391 (12)	0.321865 (12)	0.01872 (5)	
I3	1.283574 (19)	0.118445 (12)	0.272365 (13)	0.01893 (5)	
Cu1	1.07925 (4)	0.28339 (3)	0.20757 (2)	0.01739 (9)	
Cu2	0.91317 (6)	0.49932 (4)	0.10844 (4)	0.01871 (17)	0.5968 (16)
N1a	0.8219 (2)	0.88100 (15)	0.30754 (15)	0.0131 (5)	
C11	0.7234 (3)	0.96480 (18)	0.37333 (18)	0.0144 (6)	
C12	0.7803 (3)	1.0861 (2)	0.3507 (2)	0.0222 (8)	
C13	0.6638 (3)	1.1587 (2)	0.42004 (19)	0.0194 (7)	
C21	0.7404 (3)	0.76914 (19)	0.33556 (19)	0.0154 (6)	
C22	0.7369 (3)	0.69023 (19)	0.45874 (19)	0.0171 (7)	
C23	0.6214 (3)	0.5995 (2)	0.4812 (2)	0.0210 (7)	
C31	0.9801 (3)	0.85294 (18)	0.34363 (18)	0.0149 (6)	
C32	1.0882 (3)	0.7620 (2)	0.29315 (19)	0.0174 (7)	
C33	1.2416 (3)	0.7437 (2)	0.3369 (2)	0.0212 (8)	
C41	0.8411 (3)	0.93675 (19)	0.17762 (17)	0.0155 (6)	
C42	0.6926 (3)	0.9801 (3)	0.1284 (2)	0.0278 (9)	
C43	0.7306 (3)	1.0288 (2)	−0.0044 (2)	0.0243 (8)	
N2p	0.6819 (3)	0.53437 (19)	0.11898 (18)	0.0230 (7)	
C1p	0.6020 (4)	0.4452 (2)	0.1892 (2)	0.0280 (9)	
C2p	0.4469 (4)	0.4472 (2)	0.1989 (2)	0.0281 (9)	
C3p	0.3675 (3)	0.5463 (3)	0.1331 (2)	0.0286 (9)	
C4p	0.4470 (3)	0.6379 (2)	0.0616 (2)	0.0255 (8)	
C5p	0.6058 (3)	0.6306 (2)	0.0556 (2)	0.0230 (8)	
H111	0.709622	0.924955	0.458341	0.0172*	
H112	0.615337	0.975346	0.357609	0.0172*	
H121	0.788888	1.127677	0.266172	0.0267*	
H122	0.882603	1.075863	0.37637	0.0267*	
H131	0.700703	1.238077	0.407604	0.0233*	
H132	0.561853	1.169742	0.393671	0.0233*	
H133	0.652506	1.115708	0.504295	0.0233*	
H211	0.632751	0.791309	0.318782	0.0184*	
H212	0.788247	0.721678	0.279421	0.0184*	
H221	0.704259	0.740092	0.514669	0.0206*	
H222	0.841604	0.647834	0.467763	0.0206*	
H231	0.630117	0.539099	0.556794	0.0252*	
H232	0.514642	0.640855	0.484102	0.0252*	
H233	0.643691	0.559055	0.417381	0.0252*	
H311	1.030484	0.928078	0.324356	0.0178*	
H312	0.968261	0.826734	0.430099	0.0178*	
H321	1.106057	0.790999	0.206662	0.0209*	
H322	1.041211	0.684915	0.317545	0.0209*	
H331	1.307175	0.675529	0.313024	0.0255*	
H332	1.295805	0.817379	0.303006	0.0255*	
H333	1.222332	0.726026	0.423343	0.0255*	
H411	0.904564	0.879347	0.136488	0.0186*	
H412	0.907379	1.003709	0.156362	0.0186*	
H421	0.627616	0.912646	0.147852	0.0334*	
H422	0.635112	1.044646	0.162693	0.0334*	
H431	0.635019	1.06992	−0.035239	0.0292*	
H432	0.809874	1.086453	−0.024139	0.0292*	
H433	0.771582	0.96156	−0.039839	0.0292*	
H1p	0.659276	0.374425	0.236251	0.0336*	
H2p	0.391005	0.379349	0.251916	0.0337*	
H3p	0.253328	0.550155	0.13809	0.0343*	
H4p	0.391506	0.709479	0.014187	0.0306*	
H5p	0.664306	0.697485	0.003719	0.0276*	

### Atomic displacement parameters (Å^2^)

**Table:**

	*U*^11^	*U*^22^	*U*^33^	*U*^12^	*U*^13^	*U*^23^
I1	0.01901 (9)	0.01857 (7)	0.01591 (7)	−0.00176 (5)	−0.00510 (6)	−0.00505 (5)
I2	0.02250 (10)	0.01667 (7)	0.01748 (7)	0.00238 (5)	−0.00558 (6)	−0.00593 (5)
I3	0.02042 (9)	0.01691 (7)	0.02043 (7)	0.00264 (5)	−0.00814 (6)	−0.00554 (5)
Cu1	0.01693 (16)	0.01716 (12)	0.01845 (13)	0.00008 (10)	−0.00438 (11)	−0.00525 (10)
Cu2	0.0160 (3)	0.0189 (2)	0.0195 (2)	−0.00081 (17)	−0.00471 (19)	−0.00163 (17)
N1a	0.0134 (10)	0.0132 (7)	0.0128 (7)	−0.0025 (6)	−0.0018 (7)	−0.0032 (6)
C11	0.0146 (11)	0.0147 (8)	0.0134 (8)	−0.0022 (7)	0.0012 (7)	−0.0050 (7)
C12	0.0211 (14)	0.0141 (9)	0.0298 (12)	−0.0046 (8)	0.0039 (10)	−0.0074 (8)
C13	0.0239 (14)	0.0161 (9)	0.0188 (10)	−0.0013 (8)	−0.0012 (9)	−0.0073 (8)
C21	0.0153 (12)	0.0148 (9)	0.0177 (9)	−0.0054 (8)	−0.0026 (8)	−0.0051 (7)
C22	0.0209 (13)	0.0135 (9)	0.0175 (9)	−0.0043 (8)	−0.0041 (8)	−0.0029 (7)
C23	0.0192 (13)	0.0158 (9)	0.0261 (11)	−0.0068 (8)	0.0024 (9)	−0.0042 (8)
C31	0.0155 (12)	0.0140 (8)	0.0172 (9)	−0.0009 (7)	−0.0053 (8)	−0.0059 (7)
C32	0.0170 (12)	0.0184 (9)	0.0186 (9)	−0.0001 (8)	−0.0039 (8)	−0.0077 (8)
C33	0.0175 (13)	0.0243 (11)	0.0256 (11)	0.0025 (9)	−0.0080 (9)	−0.0116 (9)
C41	0.0157 (12)	0.0179 (9)	0.0112 (8)	−0.0010 (8)	−0.0006 (8)	−0.0027 (7)
C42	0.0184 (14)	0.0397 (14)	0.0193 (11)	0.0019 (11)	−0.0043 (10)	−0.0001 (10)
C43	0.0270 (15)	0.0276 (12)	0.0154 (10)	−0.0001 (10)	−0.0048 (9)	−0.0014 (8)
N2p	0.0187 (12)	0.0265 (10)	0.0237 (10)	0.0039 (8)	−0.0079 (8)	−0.0065 (8)
C1p	0.0368 (17)	0.0232 (11)	0.0230 (11)	0.0038 (10)	−0.0124 (11)	−0.0030 (9)
C2p	0.0383 (17)	0.0261 (12)	0.0217 (11)	−0.0126 (11)	−0.0096 (11)	−0.0013 (9)
C3p	0.0220 (15)	0.0369 (14)	0.0267 (12)	−0.0053 (11)	−0.0081 (11)	−0.0042 (10)
C4p	0.0203 (14)	0.0252 (11)	0.0268 (12)	0.0051 (9)	−0.0068 (10)	−0.0022 (9)
C5p	0.0184 (13)	0.0252 (11)	0.0238 (11)	0.0023 (9)	−0.0056 (10)	−0.0047 (9)

### Geometric parameters (Å, °)

**Table:**

I1—Cu1	2.5737 (4)	C31—C32	1.515 (3)
I1—Cu2	2.7752 (6)	C31—H311	1.000
I2—Cu1	2.5223 (3)	C31—H312	1.000
I2—Cu2	2.6239 (5)	C32—C33	1.526 (4)
I3—Cu1	2.5200 (3)	C32—H321	1.000
Cu1—Cu2	2.8208 (5)	C32—H322	1.000
Cu2—N2p	2.023 (2)	C33—H331	1.000
N1a—C11	1.521 (3)	C33—H332	1.000
N1a—C21	1.521 (3)	C33—H333	1.000
N1a—C31	1.520 (3)	C41—C42	1.518 (4)
N1a—C41	1.518 (3)	C41—H411	1.000
C11—C12	1.522 (3)	C41—H412	1.000
C11—H111	1.000	C42—C43	1.542 (3)
C11—H112	1.000	C42—H421	1.000
C12—C13	1.527 (4)	C42—H422	1.000
C12—H121	1.000	C43—H431	1.000
C12—H122	1.000	C43—H432	1.000
C13—H131	1.000	C43—H433	1.000
C13—H132	1.000	N2p—C1p	1.345 (3)
C13—H133	1.000	N2p—C5p	1.349 (3)
C21—C22	1.522 (3)	C1p—C2p	1.359 (5)
C21—H211	1.000	C1p—H1p	1.000
C21—H212	1.000	C2p—C3p	1.398 (4)
C22—C23	1.525 (4)	C2p—H2p	1.000
C22—H221	1.000	C3p—C4p	1.368 (4)
C22—H222	1.000	C3p—H3p	1.000
C23—H231	1.000	C4p—C5p	1.391 (4)
C23—H232	1.000	C4p—H4p	1.000
C23—H233	1.000	C5p—H5p	1.000
			
Cu1—I1—Cu2	63.521 (13)	C22—C23—H233	109.5
Cu1—I2—Cu2	66.443 (13)	H231—C23—H232	109.5
I1—Cu1—I2	118.078 (12)	H231—C23—H233	109.5
I1—Cu1—I3	119.911 (13)	H232—C23—H233	109.5
I1—Cu1—Cu2	61.722 (14)	N1a—C31—C32	115.7 (2)
I2—Cu1—I3	122.009 (14)	N1a—C31—H311	109.47
I2—Cu1—Cu2	58.506 (13)	N1a—C31—H312	109.47
I3—Cu1—Cu2	165.993 (17)	C32—C31—H311	109.47
I1—Cu2—I2	108.039 (16)	C32—C31—H312	109.47
I1—Cu2—Cu1	54.757 (12)	H311—C31—H312	102.4
I1—Cu2—N2p	102.85 (8)	C31—C32—C33	109.6 (2)
I1^i^—Cu2—I1	117.966 (18)	C31—C32—H321	109.47
I1^i^—Cu2—I2	115.09 (2)	C31—C32—H322	109.5
I1^i^—Cu2—Cu1	127.61 (2)	C33—C32—H321	109.5
I1^i^—Cu2—Cu2^i^	61.601 (15)	C33—C32—H322	109.47
I1^i^—Cu2—N2p	104.91 (6)	H321—C32—H322	109.4
I1^i^—Cu2—C1p	130.33 (5)	C32—C33—H331	109.5
I1^i^—Cu2—H1p	149.99 (2)	C32—C33—H332	109.5
I2—Cu2—Cu1	55.051 (12)	C32—C33—H333	109.5
I2—Cu2—Cu2^i^	135.09 (2)	H331—C33—H332	109.5
I2—Cu2—N2p	106.60 (6)	H331—C33—H333	109.5
Cu1—Cu2—N2p	127.48 (6)	H332—C33—H333	109.5
C11—N1a—C21	107.99 (16)	N1a—C41—C42	115.60 (18)
C11—N1a—C31	109.17 (19)	N1a—C41—H411	109.47
C11—N1a—C41	111.12 (15)	N1a—C41—H412	109.5
C21—N1a—C31	111.36 (15)	C42—C41—H411	109.5
C21—N1a—C41	108.49 (19)	C42—C41—H412	109.47
C31—N1a—C41	108.72 (16)	H411—C41—H412	102.6
N1a—C11—C12	116.13 (18)	C41—C42—C43	109.5 (2)
N1a—C11—H111	109.47	C41—C42—H421	109.5
C12—C11—H112	109.47	C41—C42—H422	109.5
H111—C11—H112	101.87	C43—C42—H421	109.5
C11—C12—C13	108.40 (19)	C43—C42—H422	109.5
C11—C12—H121	109.5	H421—C42—H422	109.4
C11—C12—H122	109.47	C42—C43—H431	109.5
C13—C12—H121	109.47	C42—C43—H432	109.5
C13—C12—H122	109.5	C42—C43—H433	109.5
H121—C12—H122	110.5	H431—C43—H432	109.5
C12—C13—H131	109.5	H431—C43—H433	109.5
C12—C13—H132	109.5	H432—C43—H433	109.5
C12—C13—H133	109.47	Cu2—N2p—C1p	114.07 (17)
H131—C13—H132	109.5	Cu2—N2p—C5p	126.45 (17)
H131—C13—H133	109.5	C1p—N2p—C5p	119.2 (2)
H132—C13—H133	109.5	N2p—C1p—C2p	122.8 (2)
N1a—C21—C22	115.4 (2)	N2p—C1p—H1p	118.6
N1a—C21—H211	109.47	C2p—C1p—H1p	118.6
N1a—C21—H212	109.47	C1p—C2p—C3p	118.4 (2)
C22—C21—H211	109.47	C1p—C2p—H2p	120.8
C22—C21—H212	109.47	C3p—C2p—H2p	120.8
H211—C21—H212	102.9	C2p—C3p—C4p	119.5 (3)
C21—C22—C23	108.7 (2)	C2p—C3p—H3p	120.2
C21—C22—H221	109.47	C4p—C3p—H3p	120.2
C21—C22—H222	109.47	C3p—C4p—C5p	119.3 (2)
C23—C22—H221	109.5	C3p—C4p—H4p	120.3
C23—C22—H222	109.47	C5p—C4p—H4p	120.3
H221—C22—H222	110.3	N2p—C5p—C4p	120.8 (2)
C22—C23—H231	109.5	N2p—C5p—H5p	119.6
C22—C23—H232	109.5	C4p—C5p—H5p	119.6

Symmetry codes: (i) −*x*+2, −*y*+1, −*z*.
